# Immune-Related Thyroid Adverse Events Predict Response to PD-1 Blockade in Patients with Melanoma

**DOI:** 10.3390/cancers14051248

**Published:** 2022-02-28

**Authors:** Anna Dawidowska, Paulina Jagodzinska-Mucha, Hanna Koseła-Paterczyk, Sylwia Jaczewska, Paweł Sobczuk, Monika Chelstowska, Maria Kowalska, Honorata Badziak-Sterczewska, Jan Poleszczuk, Piotr Rutkowski, Iwona Lugowska

**Affiliations:** 1 Early Phase Clinical Trials Unit, Maria Sklodowska-Curie National Research Institute of Oncology, 02781 Warsaw, Poland; anna.dawidowska@pib-nio.pl (A.D.); sylwia.jaczewska@pib-nio.pl (S.J.); honorata.sterczewska@pib-nio.pl (H.B.-S.); 2 Department of Soft Tissue/Bone Sarcoma and Melanoma, Maria Sklodowska-Curie National Research Institute of Oncology, 02781 Warsaw, Poland; paulina.jagodzinska-mucha@pib-nio.pl (P.J.-M.); hanna.kosela-paterczyk@pib-nio.pl (H.K.-P.); pawel.sobczuk@pib-nio.pl (P.S.); piotr.rutkowski@pib-nio.pl (P.R.); 3 Department of Pathology and Laboratory Diagnostics, Maria Sklodowska-Curie National Research Institute of Oncology, 02781 Warsaw, Poland; maria.kowalska@pib-nio.pl; 4 Department of Computational Oncology, Maria Sklodowska-Curie National Research Institute of Oncology, 02781 Warsaw, Poland; jan.poleszczuk@pib-nio.pl; 5 Nalecz Institute for Biocybernetics and Biomedical Engineering, Polish Academy of Sciences, 02109 Warsaw, Poland

**Keywords:** prognosis, endocrinopathies, immune-related adverse event, melanoma, survival

## Abstract

**Simple Summary:**

We evaluated the immune-related thyroid adverse events (irTAEs) during anti-PD-1 therapy in terms of their influence on overall survival (OS) rates in melanoma. Based on data from 249 patients with metastatic melanoma and a normal thyroid stimulating hormone (TSH) at baseline, we found that during anti-PD-1 therapy, 95 patients had a TSH outside normal ranges (32 had clinical symptoms of hypothyroidism). The 3-year OS rates in patients with clinical hypothyroidism, abnormal but clinically not significant TSH, and euthyreosis were 56%, 43%, and 32%, respectively. After adjusting the Cox model for potential confounding variables, clinically significant hypothyroidism was an independent prognostic factor with HR 0.51 (95% CI 0.29–0.87).

**Abstract:**

Antibodies against programmed cell death protein-1 or its ligand (PD-(L)1) are a standard of care in melanoma; however, this treatment may cause immune-related adverse events. The aim of this study was to evaluate the immune-related thyroid adverse events (irTAEs) during anti-PD-1 therapy and analyze their influence on the overall survival rates in melanoma. We included 249 patients with metastatic melanoma treated in our institution between 2014 and 2021; the median age was 62 years (range: 17–90); 58% were males, and 37% of patients had the *BRAF* mutation. We included patients with a normal TSH at baseline and followed up with measurement of TSH levels during immunotherapy. In our group, 95 patients had a TSH outside the normal range: 63 not clinically significant and 32 with clinical symptoms of hypothyroidism. The 3-year overall survival rate was related to the irTAEs of clinical hypothyroidism, abnormal clinically not significant TSH, and euthyreosis at 56%, 43%, and 32%, respectively (*p* = 0.002). After adjusting the Cox model for potential confounding variables, clinically significant hypothyroidism was an independent prognostic factor with HR 0.51 (95% CI 0.29–0.87). In conclusion, the patients who developed clinically significant hypothyroidism requiring replacement therapy with L-thyroxin were the group who benefitted most from anti-PD-1 treatment.

## 1. Introduction

Immune checkpoint inhibitors (ICIs) are currently the most recommended therapeutics for many cancers, including melanoma. Monoclonal antibodies acting as ICIs modulate the immune system by targeting the cytotoxic T-lymphocyte-associated antigen 4 (CTLA-4) and the programmed cell death protein-1 (PD-1). In melanoma, the ICIs are used in metastatic and adjuvant settings with a confirmed improvement in overall survival rates. Monotherapy with nivolumab or pembrolizumab or in combination with ipilimumab is a current standard of care (if no contraindications are present) [1,2,3,4,5,6].

However, in patients treated with immunotherapy, there is a risk of side effects related to the extensive activation of immune cells targeting not only tumor cells but also normal tissues. The spectrum of side effects resembles autoimmune diseases and may involve almost every organ of the body. In patients receiving anti-PD-1 therapy, 80% experience side effects, with about 10% of patients experiencing serious adverse events of Grade 3/4 according to the Common Toxicity Criteria of Adverse Events, CTCAE, and in 0.3–1.3% of patients, the outcome is fatal [7]. Based on published data and clinical practice, the most common side effects are fatigue, colitis, pneumonitis, rashes and itchiness, and disturbances to hormone levels [8].

The most common endocrine immune-related Adverse Events (irAEs) include thyroiditis, hypophysitis, and, less commonly, autoimmune diabetes mellitus and primary adrenal insufficiency. Diabetes insipidus, hypoparathyroidism, and Graves’ disease occur rarely. Hypothyroidism is the most common endocrinopathy related to immunotherapy, and its incidence is reported as 13.2% with combination therapy, 7.0% with anti-PD-1 therapy, and similar rates for ipilimumab and anti-PD-L1 alone of 3.8% and 3.9%, respectively. The hyperthyroidism rates may be underestimated due to the possibility of asymptomatic TSH changes only, and additionally, in selected patients, hypothyroidism is preceded by transient hyperthyroidism (TSH conversion) [9,10,11,12].

The limitations of immunotherapy are a lack of predictive factors enabling individualization of treatment, difficulties in predicting serious adverse events (which may be present at any time of therapy), and finally, the high cost of the drugs. There are published data about the utility of the PD-L1 expression assessment, MSI-H status, and/or tumor mutational burden (TMB); however, these markers have limited value in melanoma [13,14,15]. Recent data have shown that the irAEs may correlate with the response to ICIs, but the evidence is still conflicting in melanoma patients [16,17]. The proven relationship between overall survival and irAEs is linked to rash and vitiligo; no data are solely dedicated to endocrinopathies [15,18]. Therefore, this study aims to evaluate the relationship between immune-related thyroid dysfunction on anti-PD-1 treatment and its impact on survival rates based on retrospectively collected real-world data from a single reference institution.

## 2. Materials and Methods

We retrospectively reviewed the data of patients with histologically confirmed metastatic melanoma treated between 2014 and 2021 at the Maria Sklodowska Curie National Research Institute of Oncology in Warsaw, Poland. The inclusion criteria were ICI naïve patients who had no contraindication to anti-PD-1 therapy, received no more than 10 mg of prednisone (or equivalent) orally, and previous therapies with targeted therapy or chemotherapy were allowed. Patients with hormonal supplementation due to thyroid disease being diagnosed before anti-PD-1 therapy, and patients who had a TSH outside the normal range at the baseline were excluded from the analysis to eliminate bias. In all patients, laboratory tests such as hematology, liver and renal function tests, cardiac enzymes, as well as TSH levels were obtained before treatment and followed up regularly during treatment. The TSH changes were observed over time. The immune-related Thyroid Adverse Events (ir-TAEs) were defined as a TSH outside the normal range, which was not clinically significant (NCS abnormal TSH) or a TSH outside normal range with clinical symptoms requiring L-thyroxin supplementation (CS abnormal TSH).

The patients were treated with anti-PD-1 antibodies, either nivolumab or pembrolizumab, until the loss of clinical benefit due to disease progression or unacceptable toxicity. Disease response was evaluated by physical examination and computed tomography performed every 12 weeks according to the strict regulations of the national drug program (B.59 https://www.gov.pl/web/zdrowie/choroby-onkologiczne, accessed on 5 January 2022). CTCEA criteria were used to assess the intensity of side effects related to immunotherapy. The principle of irAEs management followed the ESMO and local clinical guidelines [19]. Patient characteristics, including age, sex, systemic treatment, mutation status, metastatic lesions, brain involvement, and LDH level, are presented in Table 1.

### Statistical Analysis

Overall survival (OS), defined as the length of time from the first day of anti-PD-1 treatment until the date of death or last follow-up (censored observation), was estimated by the Kaplan-Meier method. Differences between survival curves were tested with the log-rank test. The correction for length and lead-time bias was performed to eliminate overestimated survival duration related to different time points for TSH changes. A chi-square (χ2) statistic was applied to measure how the expectations compared to the actual observed categorical data. The multivariate Cox proportional hazards model included all statistically significant factors from univariate analysis and evaluated associations between the occurrence of irTAEs and the outcome of all-cause death. IBM SPSS Statistics for Windows (Version 25.0. New York, NY, USA) was used for statistical analyses. *p* < 0.05 was considered statistically significant. The Institutional Review Bioethics Committee approved this study and confirmed that the patient’s consent to participate in this project was not required due to the retrospective nature of this study.

## 3. Results

In the study, a total of 249 advanced/metastatic melanoma patients with performance status 0–1 based on the Eastern Cooperative Oncology Group (ECOG) were admitted to monotherapy with anti-PD-1. The median age was 65 years (range: 18–90), 144 (58%) were male, and the *BRAF* mutation was present in 93 patients (37%). Anti-PD-1 monotherapy with pembrolizumab was given to 156 patients (63%); the remaining group received nivolumab; 157 patients (70%) received anti-PD-1 therapy as a first-line therapy, while the rest of the patients received this therapy after targeted therapies, ipilimumab, or chemotherapy. With the median follow-up of 16 months (range: 1–80), the 3-year OS rate in our group was 38%, with a median OS equal to 16 months (95% CI: 10–22), 171 (69%) patients died. The patients’ characteristics are summarized in Table 1.

Ninety-five patients (38%) experienced changes in TSH outside the normal range (43 patients (17%) had an increased level of TSH, and in 52 patients (21%), the level of TSH was decreased). The median time for TSH changes was 1.5 months (range: 14 days–2 years). Thirty patients who presented a decrease in TSH experienced its conversion (increasing) during further immunotherapy. The median time for conversion was 3.1 months (range: 0.7–7.3 months). In our group, 154 patients remained in euthyreosis, 53 had no clinically significant changes in TSH levels, and 32 had clinically significant hypothyroidism requiring replacement therapy with thyroid hormones (levothyroxine). In the clinical notes, there was no information about changes in thyroid gland size in patients who develop irTEAs. The median time of clinically significant hypothyroidism was 5 months of exposure to anti-PD-l antibodies. 

**Figure 1:**
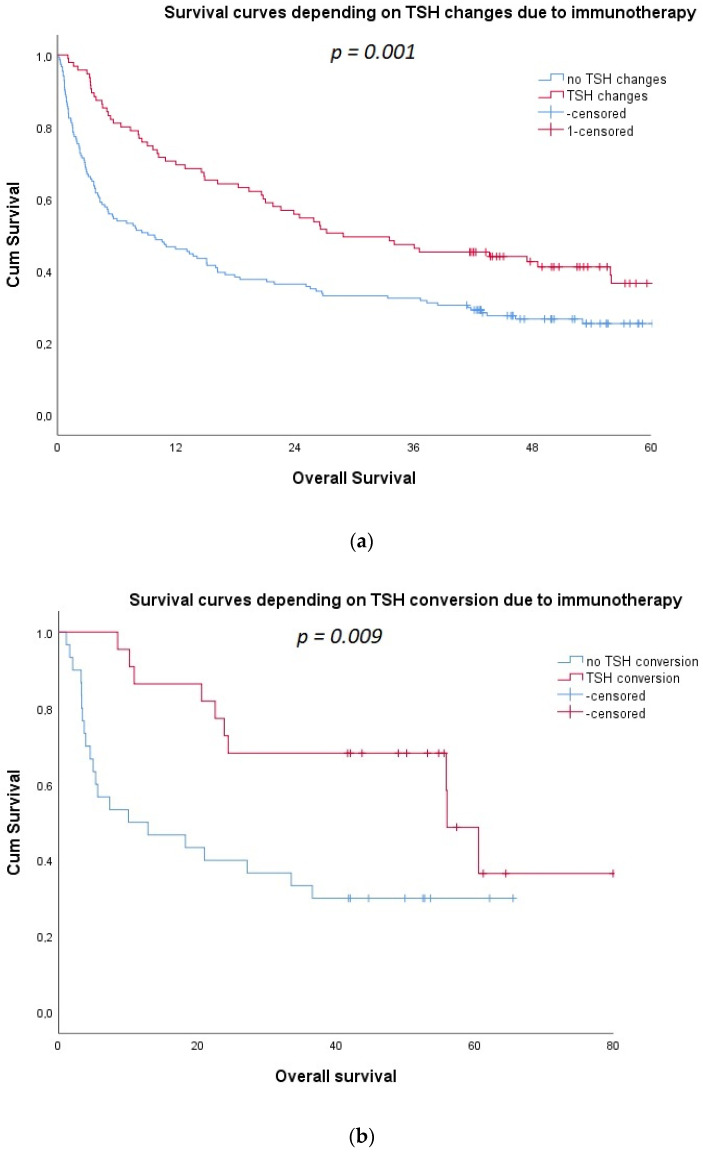

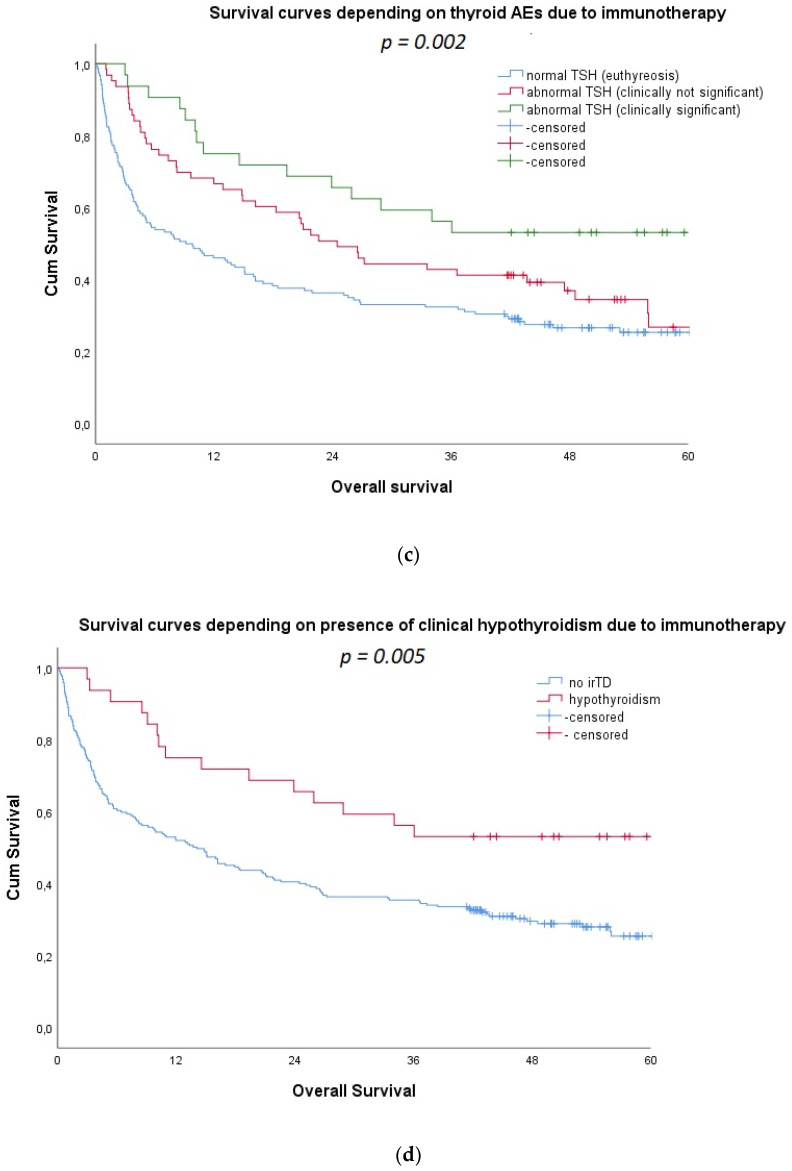

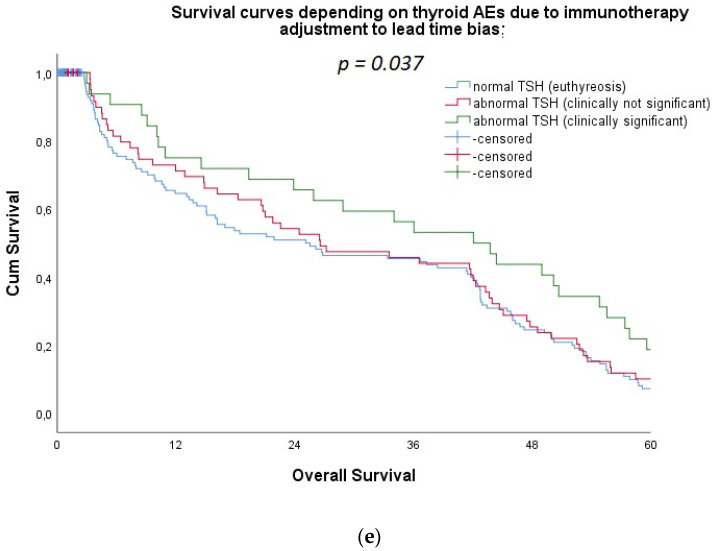
(**a**–**e**) Kaplan Meier survival curves depending on: (**a**) TSH changes, (**b**) TSH conversion, (**c**) clinical hypothyroidism, (**d**) thyroid AEs, and (**e**) thyroid AEs adjusted to the length and lead-time bias. (time provided in months).

Survival analysis showed that the 3-year OS was more favorable in patients with TSH changes vs. without changes; 47% vs. 32%. *p* = 0.001 (Figure 1a), with TSH conversion vs. without; 68% vs. 33%. *p* = 0.009 (Figure 1b), and in patients who developed clinical hypothyroidism vs. others; 56% vs. 36%. *p* = 0.005 (Figure 1c). Finally, we stratified patients’ survival according to thyroid status for clinical utility, and we observed that clinical hypothyroidism was linked with the most favorable outcome. Patients with no clinically significant changes in TSH over time had a good prognosis, but euthyreosis was a poor prognostic factor; the 3-year OS was 56%, 43%, and 32%, respectively (*p* = 0.002). The survival analyses considering the length and lead-time bias also confirmed a statistically significant relationship between irTAEs and OS regardless of timepoint of TSH changes (*p* = 0.037); (Figure 1e). The irTAEs did not cause treatment discontinuation but only careful monitoring and implementation of the recommended toxicity management. Details are presented in Table 2 and Figure 1.

The multivariate analysis with the Cox proportional hazard model adjusted to factors with an impact on prognosis from the literature and our analysis (Table 1) confirmed the significant prognostic role of irTAEs related to anti-PD-1 therapy. We also confirmed that other clinical factors such as metastases in CNS, LDH, the burden of disease, and patient’s age at diagnosis of metastatic disease had an impact on survival. Adding euthyreosis as a reference stratum, symptomatic hypothyroidism requiring hormone replacement therapy was confirmed as a favorable prognostic factor; HR 0.55 (95% CI 0.33–0.94), but the asymptomatic elevation of TSH level was not statistically significant in the Cox model. Details of the multivariate analysis are presented in Table 3.

## 4. Discussion

Here, we presented the first analysis showing a thyroid irAE as a prognostic factor in patients with melanoma treated with anti-PD1 monotherapy. Moreover, based on TSH changes due to the PD-1 blockade, we observed thyroid dysfunctions in 38% of patients, and 1/3 of those patients had clinical symptoms of irTAEs. The incidence was significantly higher than in published data from the pivotal phase III studies with pembrolizumab (10%) [20] and nivolumab (6%) [21]. In the previous real-world evidence studies, irAE were reported for 10–18% of patients [19,22,23]. Different diagnostic criteria can cause the difference since, in our study, we defined a thyroid irAE as any TSH level outside of normal ranges. The disadvantage of the limited reproducibility of CTCEA depends on the lack of objectiveness; in clinical trials, the assessment of thyroid dysfunction was based on CTCEA criteria, the presence of clinical symptoms, or the need for thyroxine supplementation. When comparing the rates of clinically relevant hypothyroidism, the incidence of 13% in our study is in line with previous reports [19,22,23]. 

Our univariate analysis showed that the 3-year OS was more favorable in patients with TSH changes than patients with a TSH in the normal range; 47% vs. 32%; *p* = 0.004. A similar correlation was observed for TSH conversion and clinical hypothyroidism, which additionally was confirmed in a multivariate analysis (HR 0.51, 95% CI 0.29–0.87). Our cohort showed that the predictive value of thyroid irAEs was severity dependent. The patients who developed hypothyroidism with a need for replacement therapy had better outcomes on anti-PD1 immunotherapy compared to the group with only abnormal TSH changes. Current evidence on the correlations between an excessive immune response, defined by the occurrence of immune-related adverse events, and the survival of patients with melanoma and anti-PD-1 therapy is inconsistent. 

Our findings are comparable to a recently published meta-analysis investigating irAE and the efficacy of ICIs, proving that occurrence of irAEs was significantly associated with higher efficacy in cancer patients, particularly endocrine, skin, and low-grade irAEs [24]. Suo et al. suggested that irAEs may be a manifestation of a patient’s ability to mount a systemic immune response from PD-1-directed therapies, which may be associated with therapeutic benefits [23]. They reported that the OS rate was significantly higher in patients who developed irAE (HR 0.46; *p* = 0.001); however, multivariable analysis confirmed only grade ≥3 irAEs as positive prognostic factors (HR, 0.29, *p* = 0.024). Similar data were presented by Indini et al., who conducted a retrospective analysis of 173 patients with metastatic melanoma treated with anti-PD1 antibodies. In their study, irAE was observed in 59% of the patients and correlated with favorable progression-free survival (PFS) and OS. In a sub-analysis of various irAEs, only vitiligo was associated with a trend toward a nonsignificant improved OS rate compared to other irAEs. Clinical hypothyroidism was observed only in 14 patients, and due to the small sample size, specific survival analyses in this cohort were not applicable [15]. The data confirming the role of immune-related endocrinopathies related to ICI in the Asian population were verified by Wu et al. in a retrospective study of 49 melanoma cases. The patients who experienced either skin/vitiligo or endocrine irAEs had longer PFS and OS than those without irAEs [25]. Other small studies have also come to similar conclusions, additionally evaluating other laboratory parameters such as LDH or the neutrophils–lymphocyte ratio [26]. 

In contrast, a large retrospective study analyzed the outcomes of 576 melanoma patients pooled from several studies treated with nivolumab, which showed that treatment-related selected AEs of any grade were associated with higher ORR; no difference in PFS was found between patients with or without irAEs [5]. In our group of patients, we did not analyze PFS since this outcome in the real world is impeded by clinical decisions beyond progression [27], and different criteria of progression may be used in routine practice. Thus, overall survival rate has been chosen in our study as the most reliable end-point for immunotherapy outcomes. In oligometastatic disease progression, a significant proportion of patients were effectively treated with local therapy such as stereotactic radiotherapy or surgical excision. Unacceptable toxicity and/or the lack of clinical benefits led to the end of treatment. 

Moreover, data from studies of other cancer types treated with immunotherapy confirm the correlations between irAEs and survival. A pooling analysis of 12 randomized controlled trials of 3815 metastatic head and neck and lung cancer patients treated with ICIs showed a significant correlation between endocrine irAEs and OS (*p* = 0.019) [28]. Other reports demonstrated that thyroid dysfunction was related to improving survival rates on pembrolizumab in NSCLC [29,30,31]. 

An observation concerning the time of onset of thyroid AEs similar to our study was presented by Freemen-Keller et al.; clinically immune-related hypothyroidism occurred in 10.8% of patients, with a median time to onset of 10.7 weeks from the start of therapy. In a multivariate analysis, a statistically significant correlation with OS was seen in patients with any grade of hypothyroidism (*p* = 0.117) or hyperthyroidism (*p* = 0.489) [22].

The mechanism of thyroid dysfunction on ICIs is still unclear; a biphasic course of irTAEs is caused by the acute inflammation and destruction of thyroid glands. The transient thyrotoxicosis is followed by hypothyroidism, which resembles classical thyroiditis. This specific biphasic course of thyroid dysfunction is named “TSH conversion”. In our study, a TSH conversion was associated with a two-times higher 3-year OS rate (68% vs. 33%. *p* = 0.009). A TSH conversion was observed after a median of three months. In the study presented by Freemen-Keller et al., only two cases with hyperthyroidism were presented with a median time to onset of 9.1 weeks, and one of them had a TSH conversion [22]. 

Assessment of the clinical utility of in-treatment prognostic markers, such as the occurrence of adverse events, is prone to the potential for lead-time bias, since patients treated for a longer period have a longer chance to develop such toxicities. In our study, the median time to TSH changes was 4.3 months, suggesting that it was rather early toxicity; however, individual patients developed irTAEs as a late event at the end of the second year of treatment. To mitigate the effect of lead-time bias, we performed an exploratory landmark analysis on patients treated with anti-PD-1 therapy for at least three months. The analysis confirmed significant differences toward better OS rates in patients with irTAEs.

The strength of the presented analysis is its first demonstration of irTAEs which may be an independent predictive factor of the efficacy of PD-1 blockade in melanoma patients. Although we confirmed the statistically significant association between irTAEs and overall survival, the results should be validated with larger prospective patient cohorts.

## 5. Conclusions

The presented population-based real world study in advanced melanoma showed an association between anti-PD-1 induced thyroid immune-related adverse events (irTAEs) and better patients’ outcomes. These results suggest that irTAEs may be a manifestation of a patient’s ability to mount a systemic immune response from PD-1–directed therapy resulting in a therapeutic benefit. The irTAEs need appropriate management per ESMO/institutional guidelines, essential for patients’ care and final outcomes. Concluding, TSH monitoring should be regularly tested before and during PD-1 immunotherapy for adverse event management and prognosis.

## Figures and Tables

**Table 1:** Baseline clinical characteristics and 3-year survival of non-operable stage III/IV melanoma patients who underwent PD-1 blockade treatment. (CNS–central nervous system; LDH–lactate dehydrogenase; ULN–the upper limit of normal).

Clinical Factors		Number of Patients (%)	3-Year OS	*p* Value *	TSH Normal	TSH CNS	TSH CS	*p* Value **
Sex	female	105 (42.2%)	41%	0.75	99 (64%)	29 (46%)	16 (50%)	0.30
	male	144 (57.8%)	36%		55 (37%)	34 (54%)	16 (50%)	
Age at diagnosis of metastatic disease	<40 years	21 (8.4%)	43%	0.03	14 (9%)	6 (9%)	1 (3%)	0.51
	40–75 years	171 (68.7%)	41%		109 (70%)	39 (62%)	23 (72%)	
	≥75 years	57 (22.9%)	24%		31 (20%)	18 (28%)	8 (25%)	
Burden of disease	1–2 organ(s)	152 (61%)	40%	<0.01	82 (53%)	44 (70%)	26 (81%)	0.01
	≥3 organs	97 (39%)	23%		72 (47%)	19 (30%)	6 (19%)	
Metastases in CNS	not present	174 (69.9%)	45%	<0.01	99 (64%)	48 (76%)	27 (84%)	0.04
	present	75 (30.1%)	20%		55 (36%)	15 (24%)	5 (16%)	
Line of treatment	1st line	175 (70.3%)	47%	<0.01	104 (68%)	48 (76%)	23 (72%)	0.44
	≥2nd line	74 (29.7%)	17%		50 (32%)	15 (24%)	9 (28%)	
Systemic regimen	Nivolumab	83 (33.3%)	42%	0.40	54 (35%)	18 (29%)	11 (34%)	0.65
	Pembrolizumab	166 (66.7%)	35%		100 (65%)	45 (71%)	21 (66%)	
*BRAF* gene	mutated	93 (37.3%)	32%	0.05	66 (43%)	15 (24%)	12 (37%)	0.31
	wildtype	156 (62.7%)	41%		88 (57%)	48 (76%)	20 (63%)	
LDH	normal range	156 (62.7%)	50%	<0.01	85 (55%)	45 (71%)	26 (81%)	0.01
	≥ULN	93 (37.3%)	16%		69 (45%)	18 (29%)	6 (19%)	

* *p* value of the log-rank test; ** *p* value of the chi^2^ test.

**Table 2:** The pattern of occurrence of TSH changes and clinical thyroid AEs.

Clinical Factors	Thyroid AEs		
TSH Changes	Present in 95 Patients (in 52 TSH Decreased)		Absent in 154 Patients
3-y OS (* *p* = 0.001)	47% (median 28; 95% CI 12–46)		32% (median 9; 95% CI 3–15)
TSH decrease (52 patients)	TSH conversion	TSH no conversion	not applicable
	30 patients	22 patients	
3-y OS (* *p* = 0.009)	68% (median 56; 95% CI 50–62)	33% (median 10; 95% CI 1–27)	
Symptomatic hypothyroidism	present in 32 patients		absent in 217 patients
3-y OS (* *p* = 0.005)	56% (median 61; 95% CI 13–108)		36% (median 14; 95% CI 8–19)
Thyroid AEs	CS abnormal TSHSymptomatic hypothyroidism	NCS abnormal TSHAsymptomatic hypothyroidism	Euthyreosis
	32 patients	63 patients	154 patients
3-y OS (* *p* = 0.002)	56% (median 61; 95% CI 13–107)	43% (median 24; 95% CI 17–31)	32% (median 9; 95% CI 3–15)

* *p*-value represents log-rank test results.

**Table 3:** The results of the multivariate Cox proportional hazard model for overall survival and thyroid AEs.

Clinical Factors	*p*-Value	HR	95% CI for HR	
			Lower	Upper
Absence of metastases in CNS	0.02	0.67	0.48	0.95
LDH elevation	<0.01	0.43	0.32	0.59
Metastatic involvement of less than 3 organs	0.02	0.68	0.49	0.94
Immunotherapy as first-line	<0.01	0.44	0.31	0.61
Age at diagnosis of metastatic disease	<0.01	1.02	1.01	1.03
Euthyreosis	0.03			
Abnormal TSH level	0.15	0.77	0.54	1.101
Symptomatic hypothyroidism	0.01	0.51	0.29	0.87

## Data Availability

The data presented in this study are available on request from the corresponding author. The data are not publicly available due to hospital procedures.
